# The Role of Smoking in the Mechanisms of Development of Chronic Obstructive Pulmonary Disease and Atherosclerosis

**DOI:** 10.3390/ijms24108725

**Published:** 2023-05-13

**Authors:** Stanislav Kotlyarov

**Affiliations:** Department of Nursing, Ryazan State Medical University, 390026 Ryazan, Russia; skmr1@yandex.ru

**Keywords:** cigarette smoking, tobacco smoking, atherosclerosis, COPD, comorbidity, innate immune system, oxidative stress

## Abstract

Tobacco smoking is a major cause of chronic obstructive pulmonary disease (COPD) and atherosclerotic cardiovascular disease (ASCVD). These diseases share common pathogenesis and significantly influence each other’s clinical presentation and prognosis. There is increasing evidence that the mechanisms underlying the comorbidity of COPD and ASCVD are complex and multifactorial. Smoking-induced systemic inflammation, impaired endothelial function and oxidative stress may contribute to the development and progression of both diseases. The components present in tobacco smoke can have adverse effects on various cellular functions, including macrophages and endothelial cells. Smoking may also affect the innate immune system, impair apoptosis, and promote oxidative stress in the respiratory and vascular systems. The purpose of this review is to discuss the importance of smoking in the mechanisms underlying the comorbid course of COPD and ASCVD.

## 1. Introduction

Smoking is a global health problem in modern medicine [1,2]. Epidemiological studies have shown a relatively high prevalence of smoking in many countries worldwide, especially among male populations. According to the World Health Organization (WHO), the number of smokers worldwide is approximately 1.3 billion people, and smoking causes about 8 million deaths per year [3]. Smoking is a major cause of many diseases and preventable deaths worldwide, including respiratory and cardiovascular diseases, cancer, and other health problems.

Numerous studies have focused on the respiratory effects of smoking. The pioneering work of Fletcher and Peto, published in 1977, underpinned the current understanding of the role of smoking in impaired lung function and the clinical importance of smoking cessation [4]. Smoking is considered the leading cause of chronic obstructive pulmonary disease (COPD). COPD has a high prevalence and is an important cause of hospitalization, disability, and mortality worldwide. The disease also carries a heavy economic and social burden on society and nations [5,6,7]. The problem of COPD is further complicated by the fact that it rarely occurs in isolation but is often associated with several comorbidities, of which atherosclerotic cardiovascular disease (ASCVD) is an important one [8,9].

Atherosclerosis is one of the most important medical and social problems of modern society [10,11]. It is widespread and is the main pathophysiological mechanism of coronary heart disease (CHD), carotid artery lesions and ischemic cerebral stroke, as well as peripheral artery disease [12]. Atherosclerosis is characterized by endothelial dysfunction, chronic inflammation, lipid accumulation and plaque formation in the intima of the vascular wall [13]. 

COPD is associated with an increased risk of cardiovascular disease and related mortality [14,15,16]. Patients with COPD have increased arterial stiffness and arterial intima-media thickness [17]. COPD exacerbations increase the risk of cardiovascular events [18]. In turn, cardiovascular disease is one of the leading causes of death in patients with COPD [19,20]. In addition, it is often not possible to determine which of these diseases develops first in such comorbid patients. Clinical data and analysis of risk factors for COPD and atherosclerosis allow us to conclude that there are some common links between the two diseases [21,22]. In addition to the fact that both diseases develop in older age groups, they share some risk factors, including smoking. In addition, sedentary lifestyle, low socioeconomic status, and some genetic factors are important for these diseases [23,24]. At the same time, many patients with both COPD and atherosclerosis do not seek timely medical care, which contributes to disease progression and the development of complications. These patients also do not always achieve effective therapeutic cooperation, which further increases the need for a better understanding of the pathophysiology of such comorbidity.

Smoking is one of the most important modifiable risk factors for both COPD and ASCVD [25,26]. Indeed, up to 80% of patients with peripheral artery disease are current or former smokers [27]. Tobacco smoking is a significant contributor to cardiovascular disease mortality patterns [28]. Smoking is considered a key integrating factor between airway inflammation, the development of systemic inflammation and oxidative stress, and the progressive development of atherosclerosis [29]. 

Cigarette smoke contains several thousand chemicals, including oxidants and free radicals, high levels of which can exceed the organism’s antioxidant defense mechanisms [30]. Cigarette smoke has a multifaceted negative effect, causing the development of inflammation, metabolic disorders and oxidative stress not only in the lungs but also at the systemic level [31,32]. Oxidative stress plays an important role in the pathogenesis of COPD, as it is associated with apoptosis and damage of alveolar epithelium, impaired mitochondrial respiration, membrane lipid peroxidation, remodeling of extracellular matrix and impaired surfactant composition and function [33,34].

Given these findings, this review aimed to describe the role of tobacco smoking in the mechanisms underlying the comorbid course of COPD and ASCVD.

## 2. The Role of Smoking in the Pathogenesis of COPD

The development and progression of COPD are characterized by airway inflammation and subsequent damage to the lung parenchyma. Prolonged exposure to particles and gases in cigarette smoke is a major risk factor for COPD development. This process leads to epithelial cell damage and the infiltration of immune cells in the lung tissue, including macrophages and neutrophils [35,36]. 

### 2.1. Disorders of the Innate Immune System in Smoking

A growing body of evidence supports the importance of impairments of the innate immune system in the development and progression of COPD [37]. The innate immune system includes many different mechanisms in which various cells such as macrophages are involved. Smoking cigarettes led to a significant increase in the number of macrophages present in the bronchoalveolar lavage. Moreover, the number of macrophages in the airways correlates with the severity of inflammation, the degree of airflow limitation, and thus the severity of COPD [38,39]. Alveolar macrophages are key participants in the innate lung immune system [40,41]. They coordinate inflammatory reactions and directly phagocytose pathogens. It is important to note that despite the increase in the total number of macrophages in the airways in smoking and COPD, phagocytosis and the elimination of microorganisms and apoptotic cells are impaired, indicating defective functional properties of macrophages [42]. Impaired phagocytosis in COPD is considered to be an important cause of disease progression, increased bacterial colonization of the airways and, accordingly, the frequency and severity of exacerbations of the disease [43,44].

Macrophages are heterogeneous in their origin and functions. The current concept suggests several polarized phenotypes that demonstrate different roles in inflammation [45]. While the “classically activated” type (M1) of macrophages is known to have a proinflammatory role, the “alternatively activated” type (M2, including subtypes M2a, M2b, and M2c) of macrophages is considered anti-inflammatory, as their function is related to tissue repair [46,47,48]. M1 macrophages produce interleukin (IL)-1, IL-6, IL-12, and tumor necrosis factor (TNF)-α, and express the enzymes cyclooxygenase 2 (COX 2) and inducible nitric oxide synthase (iNOS), which produce nitric oxide (NO) [49]. It should be noted that this classification of macrophage subtypes is simplistic, but it allows us to evaluate the significance of the complex function of these cells and the cross-linkages between the immune system and cellular metabolism [49]. Macrophage polarization is associated with a switch in cellular metabolism. These macrophage subtypes use arginine in different ways. Pro-inflammatory M1 macrophages metabolize arginine using iNOS to produce NO, which provides cytotoxic activity of macrophages against viruses, bacteria and tumor cells [50]. At the same time, M2 macrophages metabolize arginine predominantly through arginase 1 to form ornithine, which can be used to synthesize polyamines and proline necessary for tissue repair after inflammation [51,52]. Different pathways of arginine metabolism in immune cells may correspond to different phases of inflammatory activity. Interestingly, cigarette smoke extract decreases the macrophage production of NO and reactive oxygen species (ROS) and stimulates M2 macrophage polarization [53,54]. The polarization of pulmonary macrophages toward the M2 phenotype in smoking may be mediated by activation of the Janus kinase 2/signal transducer and activator of transcription 3 (JAK2/STAT3) pathway [54]. An imbalance in the ratio of M1 and M2 macrophages and correspondingly disrupted phases in inflammation may contribute to chronic inflammation in COPD.

#### 2.1.1. Immune Mechanisms Associated with Smoking in COPD

Considering the data on the polarization of macrophages and their differential role in NO production, data on the biological functions of this mediator in the lungs and their disturbances in smoking are of interest. NO produced by various NOS isoforms plays an important and diverse physiological role in the airways. It contributes to the mucociliary function of the airway epithelium by upregulating the ciliary beat frequency, regulating epithelial ion transport, and maintaining epithelial integrity [55,56,57]. This important mechanism is part of the immune defense of the airways, which is of important clinical importance and can be impaired by smoking and COPD. However, in COPD, NO biosynthesis is impaired in the endothelium of the pulmonary arteries [58]. In contrast, airway epithelial and immune cells overexpress inducible NOS (iNOS and neuronal nitric oxide synthase (nNOS)), which contributes to increased NO production and increased inflammation [59,60]. Increased expression of iNOS and nNOS was observed in the lung tissues of COPD patients [61]. Moreover, nNOS was the main source of NO at severe stages of COPD, and iNOS was involved in its production at less severe stages of the disease. Reduced eNOS expression is observed in more severe stages of COPD, which may be linked to alveolar destruction and loss of epithelial and endothelial cells. Exposure to cigarette smoke for 3 months causes selective endothelial dysfunction in guinea pig pulmonary arteries as well as decreased eNOS expression and the proliferation of smooth muscle cells in small pulmonary vessels. These changes precede the development of emphysema [62]. In eNOS^-/-^ mice, exposure to cigarette smoke for 6 months resulted in increased pulmonary artery pressure due to vascular remodeling [63].

The lungs of patients with severe COPD have increased the expression of nNOS in alveolar epithelial cells, induced by nitrosative stress, oxidative stress, and inflammatory cytokines. This increased nNOS expression leads to the production of peroxynitrite, which in turn causes further nitrosative stress [61]. Peroxynitrite is formed by the reaction of NO with superoxide anion, which is released by inflammatory cells. Peroxynitrite has a potent inflammatory effect and activates matrix metalloproteinases (MMPs) released by inflammatory cells such as neutrophils and macrophages. MMPs contribute to emphysema by destroying the extracellular matrix of the lung parenchyma [60].

**Figure 1:**
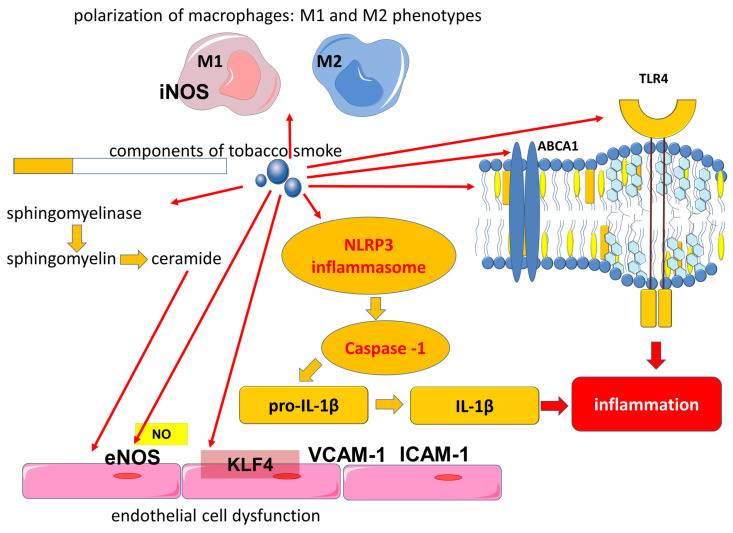
Involvement of components of tobacco smoke in the activation of proinflammatory mechanisms. Abbreviations: ABCA1—ATP binding cassette subfamily A member 1, eNOS—endothelial nitric oxide synthase, IL-1β—interleukin-1 beta, ICAM-1—intercellular adhesion molecule 1, iNOS—inducible nitric oxide synthase, KLF4—Kruppel-like factor 4, NLRP3—NLR family pyrin domain containing 3, NO—nitric oxide, TLR4—Toll-like receptor 4, VCAM-1—vascular cell adhesion molecule 1.

Another immune mechanism in which cigarette smoke is involved is related to the activation of Toll-like receptors (TLRs), which are part of the innate immune system. Toll-like receptor 4 (TLR4) recognizes lipopolysaccharides (LPS) of Gram-negative bacteria. In the lungs, TLR4 can be activated by LPS, which is associated with bacterial colonization of the airways, including exacerbations of COPD, or by exogenous oxidants. Components of tobacco smoke that include LPS are involved in the activation of TLR4 and downstream signaling pathways that contribute to cytokine production (Figure 1) [64,65,66]. Acute exposure to cigarette smoke increases TLR4 expression in the lungs of mice and rabbits. In addition, by activating TLR4, cigarette smoke increases the expression of MMP-1 in primary small airway epithelial cells in humans [67]. On the other hand, it has been shown that the functional polarization of alveolar macrophages can lead to decreased TLR2 expression in smokers and patients with COPD, which may lead to a locally impaired immune response that contributes to bacterial colonization of the airways [68].

**Figure 2:**
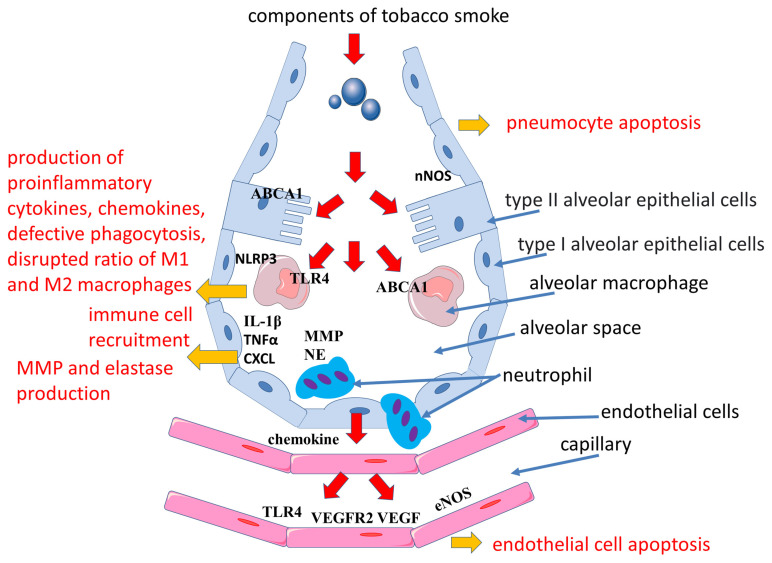
Mechanisms of emphysema development in smoking. Abbreviations: ABCA1—ATP binding cassette subfamily A member 1, CXCL—chemokine (C-X-C motif) ligand, eNOS—endothelial nitric oxide synthase, iNOS—inducible nitric oxide synthase, IL-1β—interleukin-1 beta, MMP—matrix metalloproteinases, NE—neutrophil elastase, NLRP3—NLR family pyrin domain containing 3, nNOS—neuronal nitric oxides synthase, TLR4—Toll-like receptor 4, VEGF—vascular endothelial growth factor, VEGFR2—vascular endothelial growth factor receptor 2.

In addition to TLR4 activation, cigarette smoke contributes to the activation of the signaling pathways of the NLR family pyrin domain containing 3 (NLRP3), which plays an important role in the development of COPD [69,70]. The NLRP3 inflammasome is known to be a molecular protein complex that promotes the maturation of proinflammatory cytokines such as interleukin (IL)-1β and IL-18 (Figure 1 and Figure 2). ROS are involved in the activation of the inflammasome in COPD [70]. Cigarette smoke exposure to epithelial cells in an in vitro model has been shown to promote NLRP3 inflammasome activation; furthermore, NLRP3 inflammasome activity is increased in the COPD exacerbation model [71]. 

In addition, high levels of extracellular adenosine triphosphate (eATP), which acts through binding to the purinergic receptor P2 × 7, can induce NLRP3 inflammasome activation [70,72]. eATP can be released from different cells due to cell damage or cell death and acts as a damage-associated molecular pattern (DAMP). This is consistent with the evidence that extracellular ATP accumulates in the airways in both animal models and in patients with COPD [73]. In addition, eATP was significantly elevated in the plasma of COPD patients compared with control subjects, with eATP concentrations increasing significantly with the severity of airflow limitation [74]. Moreover, smokers had higher plasma eATP concentrations compared with nonsmokers, although levels were lower than in COPD patients [74]. It is important to note that eATP causes vascular inflammation and atherosclerosis through the activation of P2Y2 [75].

Tobacco leaves and cigarette smoke condensate have previously been shown to contain tobacco glycoprotein (TGP), which is a phenol-rich glycoprotein. TGP activates the immune system, which is characterized by increased mRNA levels of IL-1α, IL-1β, IL-6 and platelet-derived growth factor (PDGF)-A in alveolar cells [76,77].

Cigarette smoke-activated macrophages released IL-1β, tumor necrosis factor-alpha (TNFα), and chemokine (C-X-C motif) ligand (CXCL), which additionally attracted monocytes, neutrophils, and lymphocytes from the bloodstream into the lungs (Figure 2). IL-1β levels were elevated in mice even during a single acute smoke exposure. Importantly, IL-1β is involved in the development of emphysema and small airway remodeling in mice, with effects comparable to TNF-alpha [78]. In turn, TNF-α is one of the most significant cytokines in COPD. Its serum levels were elevated in smokers compared to nonsmokers [79]. Elevated levels of TNF-α in lung tissue, induced sputum, and serum are found in patients with COPD [80,81,82,83]. TNF-α, also called cachexin (or cachectin), may be associated with physical frailty in COPD patients. Physical frailty is a multidimensional syndrome associated with an adverse prognosis based on skeletal muscle hypotrophy primarily of the lower extremities. In addition, TNF-α is associated with the development of emphysema in mice when exposed to cigarette smoke. TNF-α promotes the increased production of MMPs, which are involved in the development of emphysema [84]. Activated macrophages and neutrophils release proteases such as MMPs, elastases, and collagenases, which contribute to extracellular matrix damage and emphysema development [85]. In addition, systemic elevation of MMP-9 is found in COPD, which may reflect the production of MMP-9 by blood monocytes and is a marker of inflammation and may also be a predictor of decreased pulmonary function [85,86]. MMP-9 is also involved in atherogenesis, with MMP-9 levels being higher in vulnerable than in stable plaques. These data allowed us to identify MMP-9 as a predictor of atherosclerotic plaque instability and to consider its levels as a risk factor for adverse cardiovascular events in the future [87].

Another mechanism contributing to COPD exacerbations is related to the ability of cigarette smoke to enhance immune responses associated with the ingestion of viral pathogen-associated molecular patterns (PAMPs) and viruses [88]. Cigarette smoke has been found to increase airway inflammation and apoptosis induced by viral PAMP [88]. These findings support the links of viral airway infection as a cause of infectious exacerbations of COPD.

#### 2.1.2. Effects of Smoking on Lipid Metabolism and Immune System Crosslinks

It has also been shown that exposure to cigarette smoke can directly affect fatty acid metabolism in airway epithelial cells, which can influence the production of lipid mediators of inflammation and lead to changes in the ratio of saturated and unsaturated fatty acids in the phospholipids of cell plasma membranes [89]. It is important to note that the function of membrane proteins, including TLR4, is associated with the lipid composition of the macrophage plasma membranes. It has been shown that smoking can affect the lipid composition of alveolar macrophage membranes, causing a decrease in plasma membrane fluidity [90].

The effect of cigarette smoke on lipid transport is another important proinflammatory mechanism. Macrophages exposed to cigarette smoke are characterized by downregulated ABCA1 (ATP binding cassette subfamily A member 1) expression, which corresponds to impaired cholesterol efflux and inflammatory activation of macrophages, which corresponds to the upregulation of the TLR4/Myd88 pathway with the subsequent expression of MMP-9 and MMP-13 [91]. Cholesterol accumulation in macrophages is associated with their proinflammatory activation through several mechanisms, including the effect on the structural organization of cell plasma membranes and the structure of lipid rafts. Lipid rafts are specific membrane domains containing cholesterol that act as a platform for many signaling pathways, including those related to inflammation [92]. ABCA1 is a member of a large family of ABC transporters that transport various substances across cell membranes. ABCA1 exports cholesterol from the cell to the extracellular acceptor, thus participating in the maintenance of cellular cholesterol homeostasis.

It is important to note that the function of ABCA1 in alveolar macrophages is also associated with its participation in phagocytosis and efferocytosis. ABCA1 is involved in the removal of excess cholesterol formed during the uptake of apoptotic cells. A decrease in the functional activity of ABCA1, which leads to the formation of cholesterol-loaded macrophages, corresponds to their lower efficiency as phagocytes, which is consistent with the decrease in efferocytosis in the lungs of COPD patients. Thus, the lipid-transporting activity of ABCA1 is essential for normal lung function but may be impaired by smoking.

It is important to note that the transport function of high-density lipoprotein (HDL) demonstrates cross-links between metabolism and immunity, as it is one of the links for LPS utilization. 

A growing body of evidence is increasing interest in the anti-inflammatory properties of HDL, although the data are not as unequivocal. There is also conflicting evidence on the role of HDL in lung function. On the one hand, impaired lung function has been found to be associated with low HDL levels [93]. On the other hand, a negative correlation between HDL level and lung function has been shown [94]. In addition, in a large sample of adults, it was shown that patients with high HDL cholesterol levels had a greater rate of decline in forced expiratory volume in the first second (FEV1) (*p* < 0.0001) and FEV1/forced vital capacity (FVC) (*p* < 0.0001) [95]. Moreover, the rate of decline in pulmonary function in terms of effect size was comparable to the increase in the pack-years index by 10. In another study, higher HDL cholesterol levels among male adolescents were found to be associated with decreased pulmonary function (FVC and FEV1) [96]. Interestingly, higher levels of HDL may be associated with muscle condition in patients with COPD and can be considered as a biomarker of muscle volume and function [97,98].

A growing body of evidence strengthens the understanding of the importance of lipid mediators of inflammation in the pathogenesis of COPD. An imbalance between the production of proinflammatory factors and specialized pro-resolving lipid mediators contributes to the persistence of inflammation. Resolvin E1, which belongs to the group of specialized pro-resolving lipid mediators, plays an important role in preserving macrophage function during oxidative stress induced by cigarette smoke [99]. At the same time, cigarette smoke can induce an unbalanced release of lipid mediators that is characterized by a reduced prostacyclin (prostaglandin I2 or PGI2)/thromboxane A2 (TXA2) ratio, which may contribute to pulmonary vascular remodeling [100]. In this experiment, it was shown that cigarette smoke extract induced COX-2 expression while decreasing PGI2 and prostaglandin E2 (PGE2) production and increasing the production of the vasoconstrictor and proliferative mediator TXA2 [100]. Disturbances of lipid mediators’ production contribute to the development of endothelial dysfunction in COPD. While PGI2 has a protective effect on the pulmonary vasculature in response to cigarette smoke exposure, PGI2 expression is reduced in pulmonary endothelium in pulmonary emphysema [101]. Cigarette smoke extract has been shown to induce COX-2 expression in various cell types, such as endothelial cells and small airway epithelial cells [100,101,102,103]. In addition, exposure to components of cigarette smoke induces COX-2 expression in normal human lung fibroblasts with the subsequent synthesis of proinflammatory prostaglandins [104]. At the same time, it has been suggested that COX-2 may play a protective role against apoptosis in vascular endothelial cells caused by cigarette smoking [102].

### 2.2. Effect of Smoking on Apoptosis

Another important mechanism for the negative effect of cigarette smoke is its effect on apoptosis. Cigarette smoke extract induces apoptosis in mouse Ana-1 macrophages, which is accompanied by an increased release of lactate dehydrogenase, mitochondrial damage, and oxidative stress. Cigarette smoke extract also inhibited the expression of the anti-apoptotic protein Bcl-2 (B-cell lymphoma 2) and stimulated the expression of the pro-apoptotic protein Bax and Bad [105].

Cigarette smoke induced the activation of neutral sphingomyelinase 2 (nSMase2), which promotes the hydrolysis of membrane sphingomyelin to ceramides (Figure 1) [106]. Ceramides are members of a large family of sphingolipids and consist of sphingosine and various fatty acids. Ceramides can be included in the structure of the lipid bilayer of plasma membranes and also participate as a signaling molecule for apoptosis, due to which they can participate in the development of various diseases [107,108]. Increased levels of ceramides have been observed in patients with COPD, which may contribute to the development of emphysema due to their role in apoptosis [106,109,110,111]. In addition to increased ceramide levels in the lungs, the damaging effect of cigarette smoke leads to the release of ceramide-rich microparticles from the cells, resulting in increased ceramide levels in the systemic bloodstream [112]. The production of ceramide-rich microparticles during smoking is associated with the enzyme acid sphingomyelinase (aSMase), which exhibits high activity in the plasma of patients with COPD or mice exposed to cigarette smoke [112]. At the same time, elevated ceramide levels may contribute to endothelial dysfunction and coronary heart disease as well as being a prognostically unfavorable factor in cardiovascular mortality [113].

Thus, COPD is characterized by persistent chronic airway inflammation followed by bronchial remodeling, the development of airflow limitation, and increased tissue hypoxia. In addition to inflammation in the airways, the disease is characterized by systemic inflammation, the severity of which can be related to the frequency of COPD exacerbations.

### 2.3. Effect of Smoking on Endothelial Cells in COPD

Systemic inflammation and tissue hypoxia play an important role in the clinically heterogeneous course of COPD. The clinical heterogeneity of COPD underlies attempts to phenotype patients in order to improve the effectiveness of their treatment. Emphysema is an important phenotype of COPD that was described before the term COPD itself existed. In emphysema, there is destruction of the alveolar walls, resulting in a loss of alveolar surface area for gas exchange. The mechanism explaining why different patients develop emphysematous or bronchitic phenotypes in the presence of common risk factors is largely unknown. According to the vascular hypothesis, emphysema in COPD develops due to the loss of endothelial and epithelial cells by apoptosis. This concept is supported by data from histological studies of human emphysematous lungs described in 1959 by Liebow, who found that in centrilobular emphysema, the number of blood vessels in the alveolar septa is reduced [114]. The pathophysiological mechanisms of the vascular phenotype of COPD were summarized in Polverino et al. [115]. It is assumed that endothelial damage in COPD is associated with a direct toxic effect of cigarette smoke on endothelial cells, the production of autoantibodies against endothelial cells and inflammation in vessels. In addition, elevated levels of vascular oxidative stress, increased lipid peroxidation, and decreased activation of antioxidant pathways in endothelial cells are important [115]. 

Alveolar destruction has also been shown to include the apoptosis of septal endothelial cells and the decreased expression of lung endothelial vascular growth factor (VEGF) and its receptor 2 (vascular endothelial growth factor receptor 2, VEGFR2) (Figure 2) [116]. VEGF receptor signaling was found to be essential for maintaining alveolar structure, which is related to the role of VEGF in endothelial cell survival [117]. Cigarette smoke exposure has been shown to reduce VEGF and VEGFR2 levels in rat lungs and VEGF and VEGFR2 expression in the lungs of both smokers and COPD patients [118]. At the same time, exposure to cigarette smoke disrupts the VEGF165–VEGFR2 receptor signaling complex, which is an important potential mechanism of emphysema pathogenesis [118]. Moreover, in human emphysematous lungs, increased endothelial cell apoptosis corresponds to decreased expression of VEGF and VEGFR2 compared with healthy lungs [116,118,119]. However, other studies have found high levels of VEGF in smokers [120]. In addition, VEGF levels are elevated in the airways in both asymptomatic smokers and smokers with COPD [121]. Fibroblasts, which are an important source of VEGF in the lungs, have also been shown to stimulate Smad3-mediated VEGF release when exposed to cigarette smoke extract [122]. These findings suggest the heterogeneous nature of the course of COPD. It was also found that VEGF expression can vary depending on the severity of COPD [119].

Thus, pulmonary vascular remodeling in COPD is important for the clinical heterogeneity of the disease. Smoking has been shown to increase Kruppel-like factor 4 (KLF4) expression in the airway epithelium and in pulmonary vessels, contributing to their remodeling and the development of pulmonary hypertension by stimulating the proliferation of vascular smooth muscle cells [123,124]. Pulmonary hypertension is an important clinical problem in COPD that contributes to cardiac remodeling, heart failure, and is associated with a negative prognosis.

Endothelial cells play a crucial role in the innate immune response by participating in the recognition of PAMP and DAMP as well as expressing TLR4 [125,126]. Remarkably, studies have shown that TLR4 deficiency in mice led to spontaneous emphysema, without a notable increase in inflammatory cell numbers, while also triggering elevated endogenous Nox3 (NADPH Oxidase 3) activity in endothelial cells [125]. These findings highlight the significant contribution of TLR4 to the preservation of lung structural integrity and the prevention of emphysema.

Thus, COPD is a disease based on chronic persistent inflammation in the airways. Smoking, which has a multifaceted effect on various biological processes in the respiratory tract and cardiovascular system, is a significant risk factor for COPD as well as one of the mechanisms contributing to comorbidity.

## 3. Significance of Smoking in the Development of Atherosclerosis

Atherosclerosis is the result of a complex chain of processes occurring in the vascular wall, involving various cells over many years, and it includes immune and metabolic mechanisms [127,128]. The pathophysiological mechanisms of atherogenesis are closely related to the function of endothelial cells, in which a monolayer covers the arterial walls and is part of the barrier between the blood and tissues. Data obtained in recent decades have greatly expanded our understanding of the functions of endothelial cells and their role in vascular biology [129]. Many of these functions are closely related to each other and have complex pathways of regulation. The endothelium not only regulates the permeability of the vascular wall for substances and cells but is also involved in maintaining adequate hemodynamics through the production of various vasoactive substances, such as NO, and it is also involved in immune protection, coagulation regulation, and the regulation of the behavior and function of other cells [129]. 

The available evidence suggests that the endothelium plays a critical role in the early stages of atherosclerotic lesion development. Endothelial dysfunction, characterized by impaired NO bioavailability, is considered to be a key early stage of atherogenesis [130,131]. It leads to the adhesion of circulating immune cells to endothelial cells, which further contributes to the progression of atherosclerosis [132]. NO is considered one of the most important regulators of vascular hemodynamics. Nanomolar concentrations of NO promote the relaxation of vascular smooth muscle, which ensures the widening of the vascular lumen. The endothelial production of nitric oxide is associated with a specific isoform of eNOS through mechanical stimulation of endothelial cells by blood flow. The action of blood flow is detected by numerous cellular mechanosensors, which trigger a complex cascade of biochemical reactions [133].

A growing body of data strengthens the understanding of the role of impaired hemodynamics in atherogenesis. In addition to the action of systemic factors on the vascular wall, local factors such as impaired flow also play an important role in atherosclerotic lesions. It has been shown that atherosclerotic foci are more often localized in the areas of arterial branches or bends, to which disturbed blood flow and low shear stress correspond. In contrast, laminar flow and high shear stress have an atheroprotective effect. High laminar flow promoted endothelial cell elongation in the flow direction and also activated the AKT/eNOS pathway and increased the expression of eNOS and production of NO [134]. Laminar flow and high shear stress are known to promote the expression of various atheroprotective genes in endothelial cells through the induction of KLF2 and KLF4 transcription factors [135,136,137]. The exposure to cigarette smoke extract may disrupt the atheroprotective effect that high laminar flow has on endothelial function. Cigarette smoke extract increases monocyte adhesion to endothelial cells and inhibits the protective activation of the PI3K/AKT/eNOS pathway characteristic of high laminar flow [134].

Several studies have shown that cigarette smoke extract alters NO homeostasis, resulting in an impaired vasodilatory function of NO. Exposure to cigarette smoke for 32 weeks has been shown to significantly impair endothelium-dependent vasorelaxation in mice. However, in addition to the regulation of vascular tone, nitric oxide is also involved in the regulation of other biological processes, such as the regulation of leukocyte and platelet adhesion, fibrinolysis and thrombosis [138,139,140,141]. 

It is known that atherosclerosis is characterized by a decrease in eNOS expression. In contrast, the iNOS isoform contributes to an increase in NO production by other cells, which is associated with the action of proinflammatory stimuli, including cytokines and LPS. It is important to note that compared to eNOS, iNOS produces more NO, and this excessive amount of NO can have a negative effect on the arterial wall. Thus, disorders in NO production, which is at the crossroads between hemodynamics and inflammation, may play an important role in atherogenesis.

The serum of active smokers shows higher levels of TNF-α and IL-1β compared to the serum of nonsmokers as well as a greater ability to induce ROS production and COX-2 expression in endothelial cells [142]. Moreover, the simultaneous inhibition of IL-1β and TNF-α signaling pathways prevented smoking-induced endothelial dysfunction [142]. It has also been shown that in addition to increasing COX-2 expression, serum from active smokers induces the phosphorylation of p38 mitogen-activated protein kinase (MAPK) and Akt. At the same time, the activation of Akt leads to an increase in NO production, which interacts with AFC to form peroxynitrite, which can impair endothelial function [142]. In addition, peroxynitrite can also decrease the activity of prostacyclin synthase and decrease the production of prostacyclin, which is an important participant in the regulation of vascular tone [143]. It has also been suggested that NO promotes the S-nitrosylation of COX, which causes an increase in its enzymatic activity [144,145]. Increased COX activity may lead to an increased production of proinflammatory prostaglandins, which suggests a role for cyclooxygenase enzymes as important endogenous receptor targets for NO function [145,146]. 

NO production has been shown to be an important protective mechanism against endothelial damage caused by smoking. This is related to the participation of NO in the maintenance of the physiological balance between pro-apoptotic and anti-apoptotic processes in cells through participation in the modulation of some signaling molecules [147,148]. NO is involved in the S-nitrosylation of cysteine-containing proteins such as caspases and tissue trans-glutaminase, thus modulating apoptotic cell death [148,149,150]. In turn, cigarette smoke extract induces endothelial apoptosis by reducing eNOS expression and activating caspase-3 [148]. On the other hand, cytokine-induced endogenous iNOS activity may be involved in enhancing survival and maintaining endothelial function [151].

An important step in atherogenesis promoted by smoking is the increased adhesion of monocytes to endothelial cells [152]. This proinflammatory endothelial cell phenotype corresponds to increased expression of intercellular adhesion molecule 1 (ICAM-1), vascular cell adhesion molecule 1 (VCAM-1), and platelet endothelial cell adhesion molecule-1 (PECAM-1) adhesion molecules [153,154]. TNFα, which can be elevated in COPD, significantly increased the expression of intercellular adhesion molecules VCAM1, ICAM1, and E-selectin as well as the cytokines CCL2 (C-C motif ligand 2) and CX3CL1 (C-X3-C motif chemokine ligand 1) [136]. TNFα thus contributes to endothelial dysfunction. CCL2 or MCP-1 (Monocyte Chemoattractant Protein 1) is a chemokine that is a potent monocyte chemotaxis factor. It recruits immune cells, promotes local inflammation, and accelerates atherogenesis. MCP-1 expression is increased in atherosclerotic lesions and may be associated with smoking [152,155]. In addition to enhancing chemotaxis, cigarette smoke condensate induces monocyte transmigration through the endothelium, which is an important step in atherogenesis [156]. 

The key role of macrophages in the pathogenesis of atherosclerosis is well known [157,158,159]. It is believed that two main sources of macrophages in atherosclerotic lesions are monocytes originating from the peripheral blood that differentiate into macrophages and tissue-resident macrophages that develop from progenitor cells present in the adult tissues. [160]. Macrophages in atherosclerotic plaques belong to «classically activated» and «alternatively activated» subtypes, with M1 being the predominant subtype [161,162,163]. Moreover, M1 macrophages expressing proinflammatory markers are located in unstable regions, whereas M2 macrophages are predominantly in stable plaque regions [163].

Macrophages in atherosclerotic lesions are involved in the uptake and accumulation of low-density lipoproteins (LDL). Abnormal reverse cholesterol transport mediated by ABCA1 and ABCG1 may be associated with excessive cholesterol accumulation in macrophages, resulting in the formation of lipid-filled foamy cells [164,165]. In addition, excessive cholesterol accumulation can cause the proinflammatory activation of macrophages, thereby promoting inflammation in the local area [166]. These activated macrophages can produce proinflammatory cytokines, chemokines, and ROS, contributing to the maintenance of the inflammatory response. At the same time, the accumulation of foamy cells can also lead to the growth of atherosclerotic plaques. 

The ABCA1 transporter plays a crucial role in preventing atherosclerosis by facilitating the reverse cholesterol transport out of cells. However, research indicates that smoking can negatively impact the expression and functional activity of ABCA1 in individuals with CHD, leading to cholesterol accumulation in macrophages [167]. Smoking cessation in patients with CHD after 3 months resulted in increased cholesterol efflux involving ABCA1, highlighting the clinical relevance of smoking cessation [167].

An important step in atherogenesis is associated with the atherogenic modification of LDL, involving their oxidation, which makes LDL proinflammatory [158,168]. Oxidized LDL (oxLDL) induces oxidation and inflammation biomarkers in human macrophages THP-1 [169]. Individuals who smoke long-term tend to exhibit significantly higher plasma levels of LDL. Elevated levels of LDL have been shown to increase the endothelial production of oxidative markers such as malondialdehyde (MDA), inflammatory factors including TNF-α, IL-1β, and MMP-9, as well as the surface oxLDL receptor LOX-1 (Lectin-Like Oxidized Low-Density Lipoprotein Receptor 1), while decreasing endothelial eNOS and NO production [170]. Additionally, ceramides are a known atherogenic factor that can be found in higher concentrations in LDL. Ceramides enable an increased transcytosis of LDL in the endothelium, enhance the macrophage uptake of LDL cholesterol, and promote the preservation of LDL in atherosclerotic lesions [171,172,173].

As already noted, endothelial cells show some immune functions. These cells express some TLRs. At the same time, in the area of atherosclerotic lesions, an increased expression of TLR4 was detected in endothelial cells, which correlated with cell activation [174,175]. TLR4 plays a crucial role in multiple stages of atherogenesis, such as facilitating cell adhesion, enhancing macrophage uptake of oxidized lipids, and promoting the formation of “foam cells” [176,177]. Furthermore, activated endothelial cells tend to produce higher levels of proinflammatory cytokines such as IL-6, IL-8, and MCP-1 through TLR4 [178].

Thus, atherogenesis is the result of many different both local and systemic factors. Smoking affects many processes in which various cells of the vascular wall and peripheral blood flow are involved.

## 4. Comorbid Relationships of COPD and Atherosclerosis

**Figure 3:**
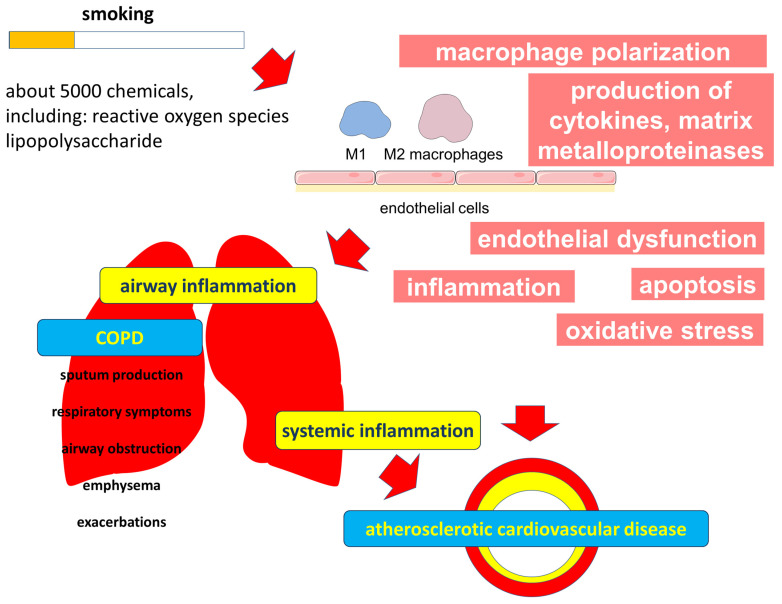
Effect of smoking on the common links in the pathogenesis of COPD and atherosclerotic cardiovascular diseases.

COPD and atherosclerosis are chronic diseases with some common risk factors, such as smoking. The exact mechanisms underlying the comorbidity of these two conditions are still debated. One possible mechanism is chronic inflammation, which is characteristic of both diseases. Systemic inflammation may contribute to the development of atherosclerosis in individuals with COPD. Another possible mechanism is oxidative stress, which is elevated in COPD and may also contribute to the development of atherosclerosis (Figure 3). 

In analyzing the comorbid associations of COPD and atherosclerosis, it is necessary to consider the clinical heterogeneity of COPD, which is not always taken into account in studies. Current data suggest that COPD is characterized by both pulmonary and extrapulmonary clinical heterogeneity, and attempts to divide patients into subtypes or phenotypes based on their clinical presentation and prognosis may help to develop more targeted approaches to patient management. However, there is still ongoing debate regarding these phenotypes. In addition to the well-known phenotypes of bronchitis and emphysema, clinicians also recognize the phenotype of COPD with frequent exacerbations, the phenotype of rapid decline in pulmonary function, the phenotype of physical frailty, and the phenotype with comorbidities, including atherosclerosis [179]. Furthermore, there is growing understanding that the clinical characteristics of COPD are related to different courses of many biochemical processes in different patients, which underlies attempts to identify disease endotypes. Thus, these and other data enhance our understanding of the complexity of both the mechanisms of COPD itself and the mechanisms linking individual disease phenotypes to atherosclerosis.

It is known that atherosclerosis is not evenly distributed among patients with COPD, and some studies have shown certain links between these diseases, including closely intertwined immune and metabolic mechanisms [180]. Analyzing these mechanisms, the “obesity paradox” in COPD seems interesting, which is known to be associated with higher mortality among patients with low body weight; conversely, a high body mass index is associated with better survival [181,182]. Decreased muscle mass and physical weakness due to skeletal muscle hypotrophy are negative factors for COPD prognosis [183]. Interestingly, the “obesity paradox” is more pronounced in patients with severe bronchial obstruction [184]. These data contradict the known notion that excess body weight is one of the most important risk factors for cardiovascular diseases. However, the coexistence of obesity with low muscle mass and impaired muscle function, known as sarcopenic obesity, is well known [185]. 

It is important to note that smoking has both short- and long-term effects on body weight [186]. In the short term, smoking can suppress appetite and increase metabolism, thereby resulting in weight loss [187,188]. It is believed that this effect is due to stimulation of the nervous system by nicotine, which leads to increased energy expenditure and decreased appetite. In addition, smoking can affect the gut microbiota, which both play a role in the regulation of body weight. Smoking is known to significantly increase the risk of developing sarcopenia [189]. An association between cigarette smoking and sarcopenia has previously been shown to be related to obesity in men [190]. Sarcopenic obesity, which develops in patients who smoke, has also been shown to progress with increasing degrees of nicotine dependence and correlates with pack/year index [191]. Simultaneously, bronchial obstruction increases with decreased muscle tissue in patients with COPD [191].

In this regard, it should also be noted that the development of emphysema may be related to nutritional deficiencies. Both historical data and data from examinations of patients with neurogenic anorexia suggest that protein malnutrition leads to emphysema development [192,193]. 

Thus, emphysema development may involve cross-metabolic and immune mechanisms that are impaired by smoking. Sarcopenia has been significantly associated with an increased risk of cardiovascular disease in men with COPD, independent of obesity and fat mass [194]. In addition, emphysema in COPD is characterized by the destruction of lung tissue due to the release of inflammatory mediators and proteases, which by acting through the systemic bloodstream may also contribute to atherosclerosis. 

Thus, the emphysematous phenotype of COPD is characterized by complex links with atherosclerosis, the search for keys to understanding which is an important research and clinical task.

These data also reinforce our understanding of the significance of physical frailty in patients with COPD. It is important to note that clinically manifested forms of atherosclerosis may decrease patients’ physical activity, which further contributes to skeletal muscle hypotrophy and increases physical weakness. In addition, the metabolic links between COPD and atherosclerosis are more extensive and include not only skeletal muscle but also nonalcoholic fatty liver disease, insulin resistance, and diabetes mellitus.

Chronic bronchitis is another known phenotype of COPD and is characterized by chronic cough and the expectoration of the sputum. This phenotype may be associated with an increased risk of atherosclerosis and cardiovascular disease [180,195]. Chronic bronchitis is associated with airway inflammation and oxidative stress, which contribute to atherosclerosis development. In addition, chronic bronchitis is associated with systemic inflammation, which can increase the risk of atherosclerosis by promoting endothelial dysfunction and accelerating plaque formation. 

The role of acute infectious exacerbations of COPD in the progression of both the disease itself and its comorbidities should be noted. In this connection, the phenotype of frequent exacerbations of COPD, which is another clinically significant variant of the disease course and is characterized by a negative prognosis, is of clinical interest. Frequent exacerbations were defined as two or more exacerbations per year. Exacerbations are characterized by worsening respiratory symptoms, such as cough, shortness of breath, and sputum production, and may be caused by viral or bacterial infections. It is important to note that COPD exacerbations are closely related to microbiota in the respiratory tract, the composition of which is disturbed by smoking. Disturbances in the composition of the airway microbiota are associated with defects in immune mechanisms and may be the cause of increased local and systemic inflammation, which underlies the comorbidity of COPD and atherosclerosis [196]. More pronounced atherosclerosis was observed in patients with exacerbation of COPD [197].

COPD exacerbations, especially those requiring hospitalization, increase the risk of myocardial infarction and stroke and require improved diagnostic approaches [18,198,199]. At the same time, smoking has been shown to be a major factor in COPD exacerbations. A high prevalence of COPD exacerbations requiring hospitalization has been noted in patients with COPD who continue to smoke [200]. Smoking cessation was associated with a decreased risk of COPD exacerbations, and the magnitude of the decreased risk depended on the duration of abstinence from smoking [201].

A phenotype with a rapid decline in pulmonary function is another clinically relevant COPD phenotype. A rapid decline in FEV1 has been shown to be clinically unfavorable because it is associated with the severity of inflammation in the airways and the progression of hypoxia. Additionally, the frequency of exacerbations may be associated with decreased pulmonary function. FEV1 is a strong predictor of all-cause mortality in patients with moderate COPD and increased cardiovascular risk [202]. Moreover, a decline in FEV1 by every 10% is accompanied by a 28% increase in the risk of cardiac death [203]. Progressive respiratory failure and intermittent hypoxia in COPD are negative factors in the development of atherosclerosis. Hypoxia can cause the post-translational modification of NO synthase, which leads to impaired NO production and contributes to atherogenesis [31]. 

It is of clinical interest to know about sex differences in the pathophysiology of COPD and atherosclerosis. Estrogens have a wide range of physiological effects, such as the regulation of oxidative stress, which has a protective effect on the cardiovascular system [204]. Interestingly, ovariectomy in non-obese women of all ages contributed to a strong association with COPD [205]. Moreover, smoking impairs the protective effects of endogenous and exogenous estrogens [206,207]. In addition, in smoking women, estrogens may be involved in the formation of toxic intermediate metabolites in the respiratory tract, which may be important in the pathogenesis of COPD [208]. These and other data have increased attention to the problem of COPD in women and the need to improve smoking cessation prevention programs.

Thus, COPD and atherosclerosis have many cross-linkages, a better study of which would improve management approaches for these patients. The current understanding of COPD phenotypes does not answer questions about all the mechanisms of comorbid relationships between COPD and atherosclerosis, but it is clear that these relationships may differ between patients (Table 1). Moreover, smoking is an important factor contributing to the initiation and progression of many of these pathological relationships.

## 5. Some Therapeutic Targets in Comorbid COPD and Atherosclerosis

Smoking cessation is an important therapeutic goal for patients with COPD and atherosclerosis. It has been shown that smoking cessation can slow the progressive decline in pulmonary function and improve quality of life and life expectancy [209,210,211].

Maintaining a healthy body weight is another important non-medical intervention for comorbid COPD and atherosclerosis [212]. The solution to this problem is closely related to the normalization of nutrition, which is also important for maintaining intestinal microbiota. The composition of the gut microbiota is associated with the development of COPD induced by cigarette smoke, and transplantation of the fecal microbiota into a mouse model of COPD reduces its development [213]. Smoking has a significant effect on the gut microbial diversity [214]. The gut microbiota of smokers has a higher proportion of Bacteroidetes and Prevotella and lower amounts of Firmicutes and Proteobacteria compared to nonsmokers [215,216]. These changes may affect the production of short-chain fatty acids (SCFAs), which, once in the systemic bloodstream, have multiple effects, including in the function of the innate lung immune system [217]. These findings support the known evidence for the lung–gut axis, in which the gut microbiota plays an important role. Studies of the gut microbiota in COPD have shown a decrease in the diversity of the microbial community compared to that in healthy individuals. COPD may contribute to the increased permeability of the intestinal mucosal barrier, which contributes to systemic inflammation.

Therefore, nutrition is an important therapeutic intervention in COPD. High-fiber intake is inversely related to the incidence of COPD in both current and former smokers [218,219]. A high-fiber diet reduced the progression of cigarette smoke-induced emphysema through the effects of fiber on gut microbiota composition and increased the production of SCFAs such as acetate, propionate, and butyrate. These SCFAs reduced the progression of emphysema and inflammation associated with cigarette smoke exposure [220]. At the same time, the intake of high-fat foods has a negative effect on the production of SCFAs and the progression of COPD. The modulation of nutrition plays an important role in the pathogenesis of atherosclerosis. Butyrate and acetate have been shown to increase NO bioavailability and improve Ang II-induced endothelial dysfunction [221]. The key mechanism through which SCFAs act on endothelial function is the reduction in ROS levels in the vascular wall, which is followed by the prevention of NO inactivation [221].

Thus, smoking cessation and nutritional modulation are important therapeutic targets that act via common immune and metabolic mechanisms.

One of the important metabolic targets in COPD and atherosclerosis is the correction of lipid metabolism disorders, which are also considered to be an important link between COPD and atherosclerosis. Elevated serum levels of oxLDL have been correlated with declining lung function, inflammation, and oxidative stress in COPD [222]. Smoking is associated with an atherogenic lipid profile, affecting both the amount and function of HDL, which is a significant mechanism for increased cardiovascular risk in smokers [223]. Various mechanisms are known to be responsible for atherogenic changes in lipoproteins, including changes in cholesteryl ester transfer protein (CETP) activity during smoking [224]. 

Given the commonality of inflammatory and metabolic mechanisms, data on the use of statins in patients with COPD, which have anti-inflammatory and antioxidant properties, are of interest [225], which have anti-inflammatory and antioxidant properties. In smokers and former smokers, statins have been shown to be associated with a slower decline in lung function regardless of underlying lung disease [226]. Statin use attenuates the decline in pulmonary function in the elderly, with the magnitude of the beneficial effect depending on smoking status [227]. Simvastatin prevents airway inflammation and epithelial damage in rat experiments when pretreated with simvastatin one week before tobacco smoke exposure [228]. Simvastatin also inhibited lung parenchyma destruction, reduced inflammatory cell infiltration and reduced matrix metalloproteinase-9 activity in rat and guinea pig lung tissue [229,230,231]. At the same time, smoking has been shown to reduce the positive effect of statins [232].

In contrast, the use of statins was not associated with the occurrence of COPD in adults, but it reduced the likelihood of exacerbations of the disease [233]. Moreover, the reduction in the risk of COPD exacerbation was significant with both the short- and long-term use of statins [234]. In addition, statin use was associated with a significant reduction in the risk of hospitalized exacerbations in patients with COPD and showed efficacy in patients with frequent exacerbations of COPD [235]. The long-term use of statins, in addition to reducing the risk of acute exacerbations of COPD, also reduces all-cause and cardiovascular mortality [236,237].

It should be noted that in other studies, statin use was not associated with the risk of COPD exacerbation [238,239]. It has been shown that simvastatin use had no effect on the frequency of COPD exacerbations [240]. In addition, an analysis of eight studies involving 1,323 people with COPD showed that statin use, although it reduced CRP and IL-6 levels, had no significant clinical benefit in patients with COPD [241]. The effect of statins on mortality in patients with COPD at 10-year follow-up has also not been shown [238].

Thus, information regarding the clinical efficacy of statins in COPD is varied. It is assumed that the benefits of statins shown in some studies are related to their effect on the main risk factors of cardiovascular diseases rather than on the pathophysiological mechanisms of COPD [242].

## 6. Conclusions

Based on the available data, it appears that tobacco smoking has a complex and negative impact on multiple immune and metabolic processes as well as on the mechanisms of oxidative stress and apoptosis. These processes play a crucial role in the comorbid development of both COPD and atherosclerosis. Smoking not only affects the respiratory tract, but it also has systemic effects. Cells such as macrophages and endothelial cells are integral to the development of these diseases, and smoking can impair many of their functions.

COPD is characterized by chronic inflammation of the airways and lung tissue, resulting in airflow limitation and impaired gas exchange. Atherosclerosis, on the other hand, is a chronic inflammatory disease of the arteries that leads to the buildup of fatty deposits and narrowing of the arterial lumen. Smoking is known to promote the development and progression of both diseases by causing systemic inflammation, endothelial dysfunction, and oxidative stress.

It should be noted that the current review has some limitations, as it only discusses the effect of smoking on certain cell types in the pathogenesis of COPD and atherosclerosis. At the same time, other cells, including cells participating in the adaptive immune system, are also involved in the pathogenesis of these diseases. A growing body of evidence strengthens the understanding of adaptive immune cells in COPD and cardiovascular diseases [243,244,245,246,247,248,249], which is a promising area for future research. In addition, the links between innate and adaptive immune systems and their dysfunction in smoking are of interest. Other important areas of research include the study of cross-linkages between immune and metabolic processes and the disruption of these links in smoking. The mechanisms of the clinically heterogeneous course of COPD and the links between different phenotypes of COPD and atherosclerosis are promising directions for future research. The search for diagnostic and therapeutic tools that could influence impaired immune and metabolic mechanisms in COPD smoking is also clinically important.

Thus, COPD and ASCVD are diseases that develop over many years and have some common links in pathogenesis, a better understanding of which would improve diagnostic efficiency and the quality of treatment. Smoking cessation is an important medical goal that will prevent the development of the diseases or improve their clinical outcomes.

## Figures and Tables

**Table 1:** Current limitations of clinical research on comorbidity.

Factors Complicating Clinical Studies on the Role of Smoking in the Comorbid Course of COPD and Atherosclerosis
1. The diagnosis of COPD is mainly based on spirometry data.
2. Impossibility of determining disease onset.
3. It is difficult to consider the role of other COPD risk factors such as particulate matter and the presence of COPD in nonsmokers.
4. Different models of the disease course and progression.
5. Clinical heterogeneity of COPD: different phenotypes and endotypes of the disease are known.
6. Presence of other clinically significant comorbidities.
7. Effect of pharmacotherapy on study results.
8. Complex cross-linkages of the innate immune system and metabolism.

## Data Availability

Not applicable.
